# Integrated and Total HIV-1 DNA Predict *Ex Vivo* Viral Outgrowth

**DOI:** 10.1371/journal.ppat.1005472

**Published:** 2016-03-03

**Authors:** Maja Kiselinova, Ward De Spiegelaere, Maria Jose Buzon, Eva Malatinkova, Mathias Lichterfeld, Linos Vandekerckhove

**Affiliations:** 1 HIV Translational Research Unit (HTRU), Department of Internal Medicine, Ghent University and Ghent University Hospital, Ghent, Belgium; 2 Ragon Institute of MGH, MIT and Harvard, Boston, Massachusetts, United States of America; 3 Infectious Disease Division, Massachusetts General Hospital, Boston, Massachusetts, United States of America; 4 Infectious Disease Division, Brigham and Women’s Hospital, Boston, Massachusetts, United States of America; University of North Carolina at Chapel Hill, UNITED STATES

## Abstract

The persistence of a reservoir of latently infected CD4 T cells remains one of the major obstacles to cure HIV. Numerous strategies are being explored to eliminate this reservoir. To translate these efforts into clinical trials, there is a strong need for validated biomarkers that can monitor the reservoir over time *in vivo*. A comprehensive study was designed to evaluate and compare potential HIV-1 reservoir biomarkers. A cohort of 25 patients, treated with suppressive antiretroviral therapy was sampled at three time points, with median of 2.5 years (IQR: 2.4–2.6) between time point 1 and 2; and median of 31 days (IQR: 28–36) between time point 2 and 3. Patients were median of 6 years (IQR: 3–12) on ART, and plasma viral load (<50 copies/ml) was suppressed for median of 4 years (IQR: 2–8). Total HIV-1 DNA, unspliced (us) and multiply spliced HIV-1 RNA, and 2LTR circles were quantified by digital PCR in peripheral blood, at 3 time points. At the second time point, a viral outgrowth assay (VOA) was performed, and integrated HIV-1 DNA and relative mRNA expression levels of HIV-1 restriction factors were quantified. No significant change was found for long- and short-term dynamics of all HIV-1 markers tested in peripheral blood. Integrated HIV-1 DNA was associated with total HIV-1 DNA (p<0.001, R² = 0.85), us HIV-1 RNA (p = 0.029, R² = 0.40), and VOA (p = 0.041, R^2^ = 0.44). Replication-competent virus was detected in 80% of patients by the VOA and it correlated with total HIV-1 DNA (*p =* 0.039, R² = 0.54). The mean quantification difference between Alu-PCR and VOA was 2.88 log_10_, and 2.23 log_10_ between total HIV-1 DNA and VOA. The levels of usHIV-1 RNA were inversely correlated with mRNA levels of several HIV-1 restriction factors (TRIM5α, SAMHD1, MX2, SLFN11, pSIP1). Our study reveals important correlations between the viral outgrowth and total and integrated HIV-1 DNA measures, suggesting that the total pool of HIV-1 DNA may predict the size of the replication-competent virus in ART suppressed patients.

## Author Summary

Current HIV-1 research aims to find a cure for HIV-1, either by pursuing viral eradication or by attempting to attain an immune-mediated functional cure. For the purpose of interpreting the findings of these eradication strategies, a validated representative biomarker of the replication-competent latent HIV-1 reservoir is urgently needed. In this study we have evaluated several cell-associated HIV-1 persistence markers, and we have measured replication-competent reservoir using the viral outgrowth assay (VOA). The results show a correlation between the pool of HIV-1 DNA and the replication-competent reservoir. Our data show that the pool of HIV-1 DNA (total or integrated HIV-1 DNA) can predict the amount of replication-competent latent HIV-1 in patients receiving treatment. Hence, PCR based assays quantifying integrated and/or total HIV-1 DNA can play an important role in future studies aiming at HIV-1 eradication.

## Introduction

Current antiretroviral therapy (ART) successfully suppresses viral replication and reduces HIV-1 transmission, but fails to completely eliminate HIV-1 [1]. Even after long-term ART, a latent reservoir of HIV-1 DNA persists and remains fully competent to refuel viral rebound when ART is stopped [2, 3]. An increasing number of strategies to target the latent HIV-1 reservoir are being pursued and initial patient clinical trials have been performed [4]. However, interpretation of clinical data is hampered due to the lack of validated biomarkers that can monitor the size of the replication-competent reservoir.

Different markers to monitor changes in the size and composition of the viral reservoir are being explored [5]. In this context, it is crucial to clearly define the characteristics of the reservoir that causes viral rebound upon treatment discontinuation. It is well known that only a small fraction of integrated HIV-1 DNA is composed of intact HIV-1 DNA that is capable to produce replication-competent virus. This small fraction of replication-competent virus is the sole contributor to viral persistence and sole source of viral rebound after treatment discontinuation. Hence, an effective biomarker needs to correlate with the changes in the fraction of the replication-competent reservoir and should not be biased by the levels of archival, dead-end integrated proviruses [5].

The existence of the replication-competent viral reservoir was first observed by experiments in which latent provirus was reactivated after stimulation of isolated patient CD4+ T cells using the so-called viral outgrowth assay (VOA) [5, 6]. VOAs have now been designed to quantify the replication-competent viral reservoir by reactivating multiple replicates at a limiting dilution [6]. The VOA has long been considered as the gold standard for measuring the HIV-1 reservoir in patients. However, recent data indicate that the VOA underestimates the actual viral reservoir, as consecutive reactivation of the initially negative cells often induces reactivation of virus which was not reactivated in the first round of stimulation [7]. This indicates that the total number of intact viral sequences outnumbers the amount of virus that becomes reactivated [7]. Therefore, it remains to be assessed whether the VOA outcome correlates with the actual replication-competent viral reservoir *in vivo*. In addition, practical issues, such as time to result, assay costs and the large amount of blood required may impede the implementation of VOA assays in a clinical setting.

Alternative markers, based on quantitative PCR of total or integrated HIV-1 DNA have also been explored [8]. These markers cannot discern dead-end integrated HIV-1 DNA from the replication-competent reservoir. PCR-based markers overestimate the reservoir and this may obscure changes in the amount of replication-competent HIV-1 DNA [5]. Despite these drawbacks, PCR based markers are the most commonly used tool in HIV reservoir research. These assays combine a high sensitivity with a proficient accuracy. They are also cheap, easy to implement and can be performed for high throughput analysis. Interestingly, recent results indicate that measuring total and integrated HIV-1 DNA may be clinically relevant. For instance, total HIV-1 DNA was found to inversely correlate to the time to viral rebound in early treated patients interrupting ART [9, 10]. In addition, markers for viral reservoir dynamics, such as viral replication or mRNA transcription may also be promising tools to assess the effect of anti HIV-1 latency interventions. Episomal 2LTR circles are formed as a transient by-product of failed HIV-1 DNA integration, indicating recent production of new infective virus from the reservoir [11]. Proviral transcription is quantified by measuring the amount of cell-associated (CA) RNA [12, 13]. The levels of unspliced CA HIV-1 RNA are associated with virological failure in patients on treatment [11, 14].

A thorough validation of HIV-1 persistence markers in clinically relevant samples will be needed to identify the optimal biomarker for HIV-1 reservoir research. This will require a comprehensive evaluation and comparison of candidate markers, and in the future, an assessment of these markers in treatment interruption studies. Moreover, a critical comparison of the quantitative agreement of the methods should be implemented, as a high correlation does not automatically imply that there is good quantitative agreement [15]. To date, only limited studies have compared PCR-based assays with the VOA. An initial study by Eriksson et al., revealed no correlation with HIV-1 DNA or 2LTR circles, but a significant association was observed between integrated HIV-1 DNA and the VOA outcome [12]. In addition, a study of Buzon et al., confirmed a significant correlation between VOA and HIV-1 DNA in CD4+ T-cells [16]. However, the levels of cell-associated HIV-1 RNA have not been compared to the VOA outcome yet.

Here, we performed a comprehensive evaluation of viral reservoir markers quantified by digital (dPCR) and real time PCR (qPCR) and by VOA. Subsequently, a Bland Altman analysis was performed to evaluate the quantitative agreement between the methods. In addition, this study aimed at evaluating both long- and short-term variation of PCR based reservoir markers in order to guarantee that differences observed in intervention trials are genuine rather that assay variations. We used 25 patient samples, selected from a previous study with the purpose of having patients with varying levels of HIV-1 burden, representing the actual variation observed in clinical samples. Moreover, the gene expression of several known HIV-1 restriction and host factors was quantified to evaluate if these are correlated with any of the reservoir markers.

## Results

### Characteristics of the study population

The majority of patients were males (19/25, 76%), with a median age of 48 years (IQR: 44–61). Current CD4+ T count was median of 507 cells/μl (IQR: 420–642), and CD4+ T nadir count of median 195 cells/μl (IQR: 118–260). The pre-ART plasma viral load was median of 5.0 log_10_ copies/ml (IQR: 4.7–5.6). All patients were receiving ART at the time of sampling, with median of 6 years (IQR: 3–12) and a viral load suppression (<50 copies/ml) of median 4 years (IQR: 2–8). Patients had residual plasma HIV-1 RNA of median 4 copies/ml (IQR: 1–7) plasma, which correlated well only with the baseline measurement for unspliced HIV- RNA (p = 0.028, R^2^ = 0.40). About half of the patient cohort was infected with subtype B virus (52%), 20% with non-B subtype and for the others (28%) the viral subtype was unknown.

### Long- and short-term dynamics of intracellular markers of HIV-1 persistence in blood

**Fig 1:**
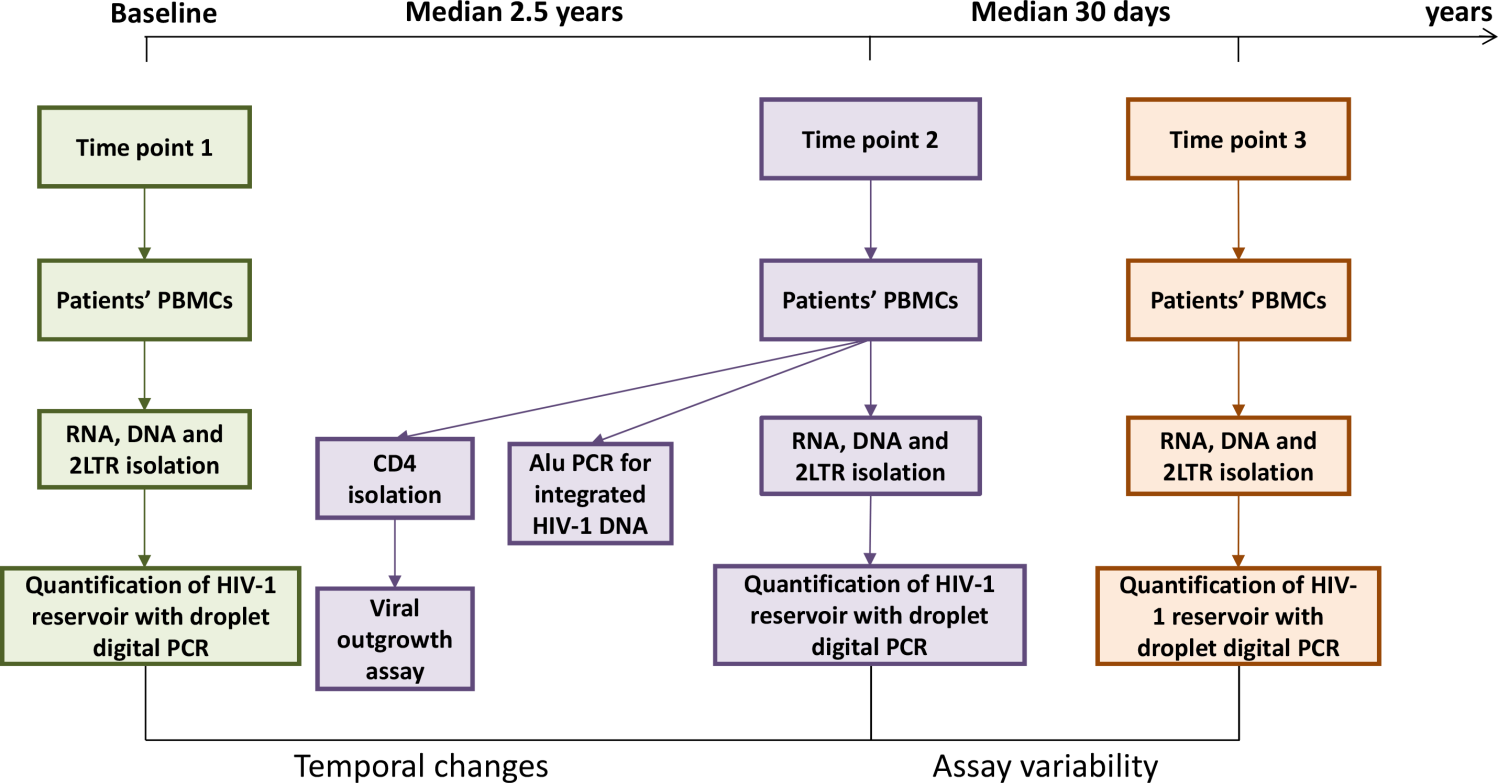
Workflow schematics.

Measured markers of HIV-1 persistence in blood included total HIV-1 DNA, unspliced and multiply spliced HIV-1 RNA, and 2-LTR circles in PBMCs of all patients at three different time points. The time difference between baseline and second measurement was a median (IQR) of 2.5 years (2.4–2.6), and the time difference between the second and third measurement was a median (IQR) of 31 days (28–36) (Fig 1 workflow).

Unspliced HIV-1 RNA was detected in the majority of subjects at each time point (92%, 72%, 80%, respectively); with median log_10_ copies/10^6^ CD4+ T cells of 1.3 (IQR: 0.9–1.7); 1.0 (IQR: 0.8–1.5) and 1.2 (IQR: 0.6–1.5) at the three time points, respectively. Multiply spliced HIV-1 RNA was detected in 74%, 64%, 68% of patients at each time point, respectively; with median log_10_ copies/10^6^ CD4+ T cells of 0.6 (IQR: 0.4–0.9); median 0.5 (IQR: 0.3–0.7); and median 0.5 (IQR: 0.3–0.5) at the three time points, respectively. Total HIV-1 DNA was detected in all subjects at each time point; with median log_10_ copies/10^6^ CD4+ T cells of 3.0 (IQR: 2.2–3.6), 3.4 (IQR: 2.6–3.7) and 3.1 (IQR: 2.4–3.6) at the three time points, respectively. 2LTR circles were detected in 52%, 56%, 76% patients at each of the three time points, respectively, with median log_10_ copies/10^6^ CD4+ T cells of 0.6 (IQR: 0.7–1.3), 0.5 (IQR: 0.4–0.7) and 0.5 (IQR: 0.4–0.6) at the three time points, respectively.

**Fig 2:**
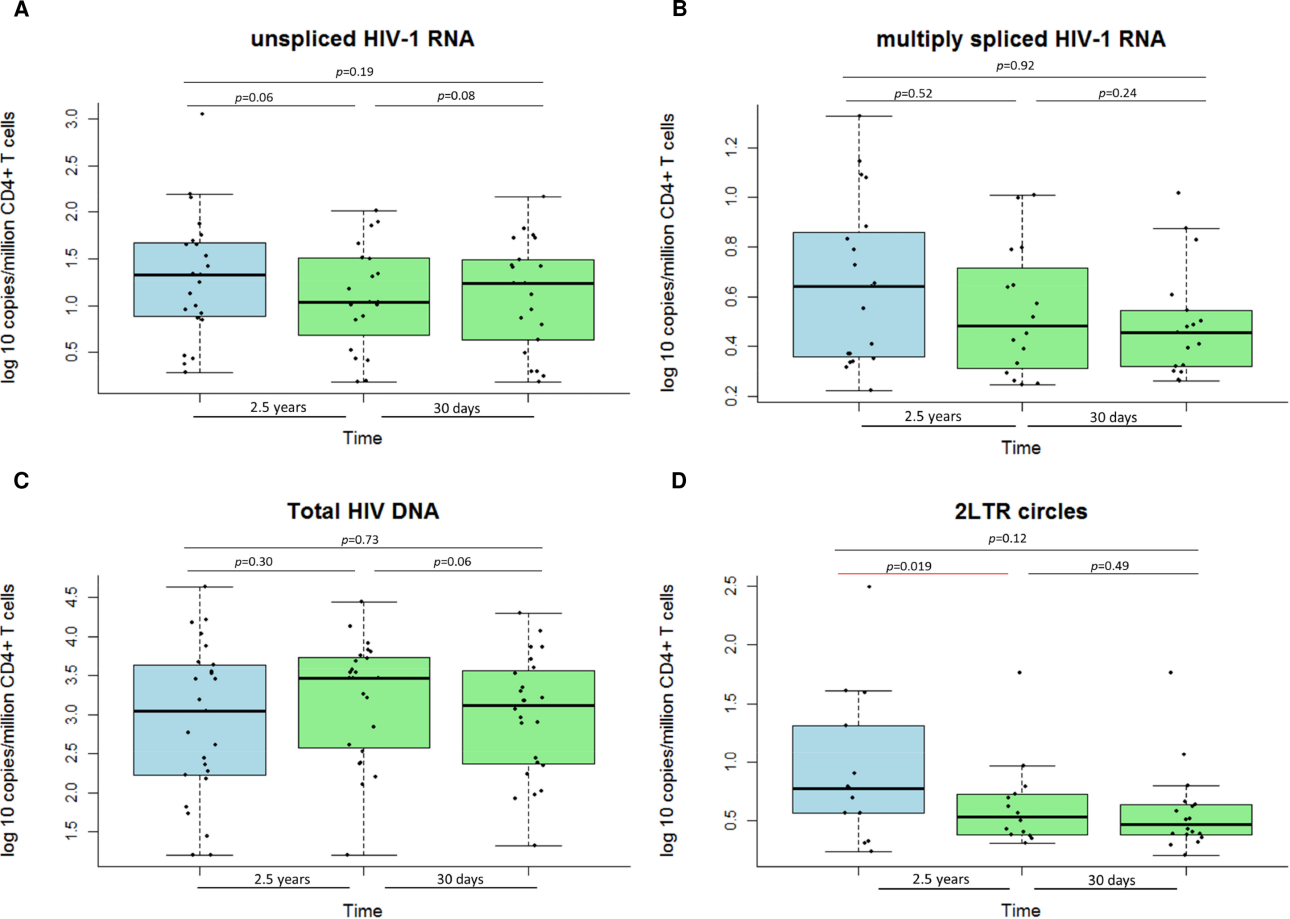
Viral reservoir dynamics. Long and short-term dynamics of cellular markers of HIV-1 persistence. A) unspliced HIV-1 RNA; B) multiply spliced HIV-1 RNA; C) Total HIV-1 DNA; and D) 2LTR circles. *Of note: all analysis was performed using non-parametric statistics.

The long-term (median of 2.5 (IQR: 2.4–2.6) years) and the short-term (median of 31 (IQR: 28–36) days) viral reservoir dynamics of unspliced, multiply spliced HIV-1 RNA, and total HIV-1 DNA did not significantly differ over time (Fig 2A–2C). Except for 2LTR circles, where a significant decrease was observed for the long-term reservoir dynamics (Fig 2D).

The inter-assay coefficient of variation for total HIV-1 DNA was a median of 13% (IQR: 7–26) for unspliced HIV-1 RNA; the median was 34% (IQR: 27–43) for multiply spliced HIV-1 RNA, 6% (IQR: 3–12) for total HIV-1 DNA and 26% (IQR: 17–35) for 2LTR circles. Test-retest variability within patients for quantification of unspliced HIV-1 RNA was a median of 22% (IQR: 17–28) with R^2^ = 0.92, a median 47% (IQR: 30–83) with R^2^ = 0.72 for multiply spliced HIV-1 RNA, a median of 6% (IQR: 6–7) with R^2^ = 0.99 for total HIV-1 DNA, and a median of 34% for 2LTR circles (IQR: 16–86) with R^2^ = 0.95 (S1A–S1D Fig).

### HIV-1 reservoir size and its potential for reactivation

Biomarkers for estimating the size of the HIV-1 viral reservoir and its potential to be reactivated included integrated HIV-1 DNA and VOA. Both were assessed at the second time point. Integrated HIV-1 DNA was detected in all patients with median (IQR) of 3.8 (3.5–4.7) log_10_ copies/10⁶ CD4+ T cells. Of note, the fact that integrated HIV-1 DNA copies detected by Alu PCR were higher than total HIV-1 DNA reflects the differences in assay standardization. For each infected cell, the distance to the nearest Alu element is different. Therefore, a correction factor is applied based on an integration standard containing proviruses integrated at different distances from the nearest Alu element. Thus, the Alu PCR is a real-time PCR based assay, where a standard curve is used that provides a correction factor. Whereas the quantification for total HIV-1 DNA was performed on digital PCR where no standard curve is used. Hence, the absolute values for both integrated HIV DNA and total HIV DNA cannot be directly compared. The principle of this assay has been previously described by us and others [17, 18].

**Fig 3:**
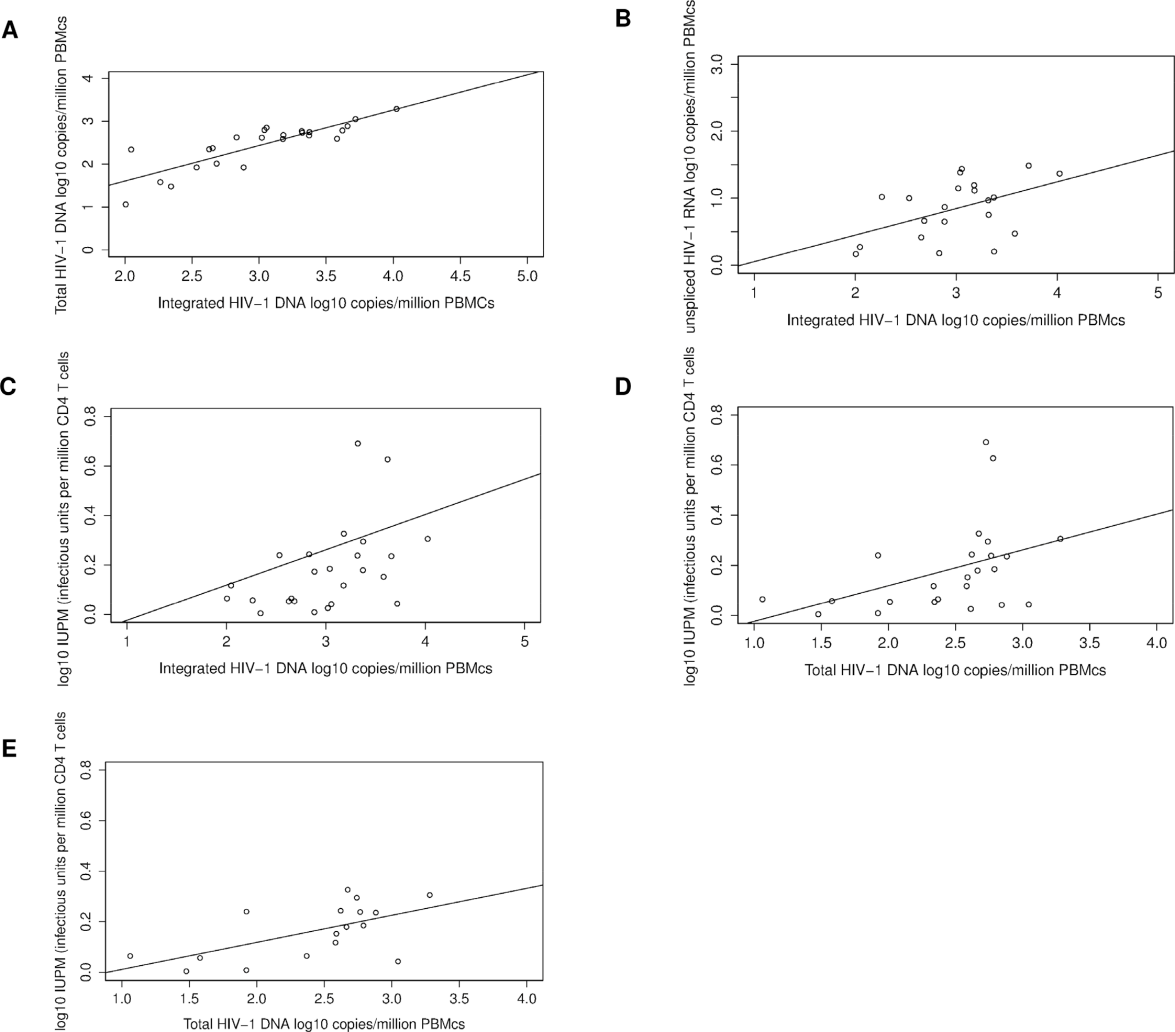
Linear regression analysis between viral reservoir markers. Associations between: A) Integrated HIV-1 DNA and total HIV-1 DNA; B) Integrated HIV-1 DNA and unspliced HIV-1 RNA; C) Integrated HIV-1 DNA and viral outgrowth assay; D) Total HIV-1 DNA and viral outgrowth assay; and E) Total HIV-1 DNA and viral outgrowth assay–outliers removed.

Integrated HIV-1 DNA was associated with total HIV-1 DNA (p<0.001, adjusted R² = 0.71, Spearman’s correlation coefficient R^2^ = 0.85) (Fig 3A), unspliced HIV-1 RNA (p = 0.029, adjusted R² = 0.20, Spearman’s correlation coefficient R^2^ = 0.40) (Fig 3B) and with the viral outgrowth assay (p = 0.041, adjusted R^2^ = 0.22, with Spearman’s correlation coefficient R^2^ = 0.44) (Fig 3C).

Reactivated replication-competent virus was detected in 20/25 (80%) patients by VOA with a median of 0.52 (IQR: 0.16–0.81) infectious units/10⁶ CD4+ T cells. Total HIV-1 DNA was associated with IUPM (p = 0.039, adjusted R² = 0.20, Spearman’s correlation coefficient R^2^ = 0.54) (Fig 3D). Replication-competent HIV-1 was recovered significantly more frequently from patients with total HIV-1 DNA higher than 3.1 log_10_ copies/10^6^ CD4 + T cells (p = 0.04). The significant association remained after the removal of the two samples with highest value for replication-competent virus, assessed by linear correlation analysis (p = 0.011, adjusted R^2^ = 0.32, Spearman’s R^2^ = 0.53) (Fig 3E).

**Fig 4:**
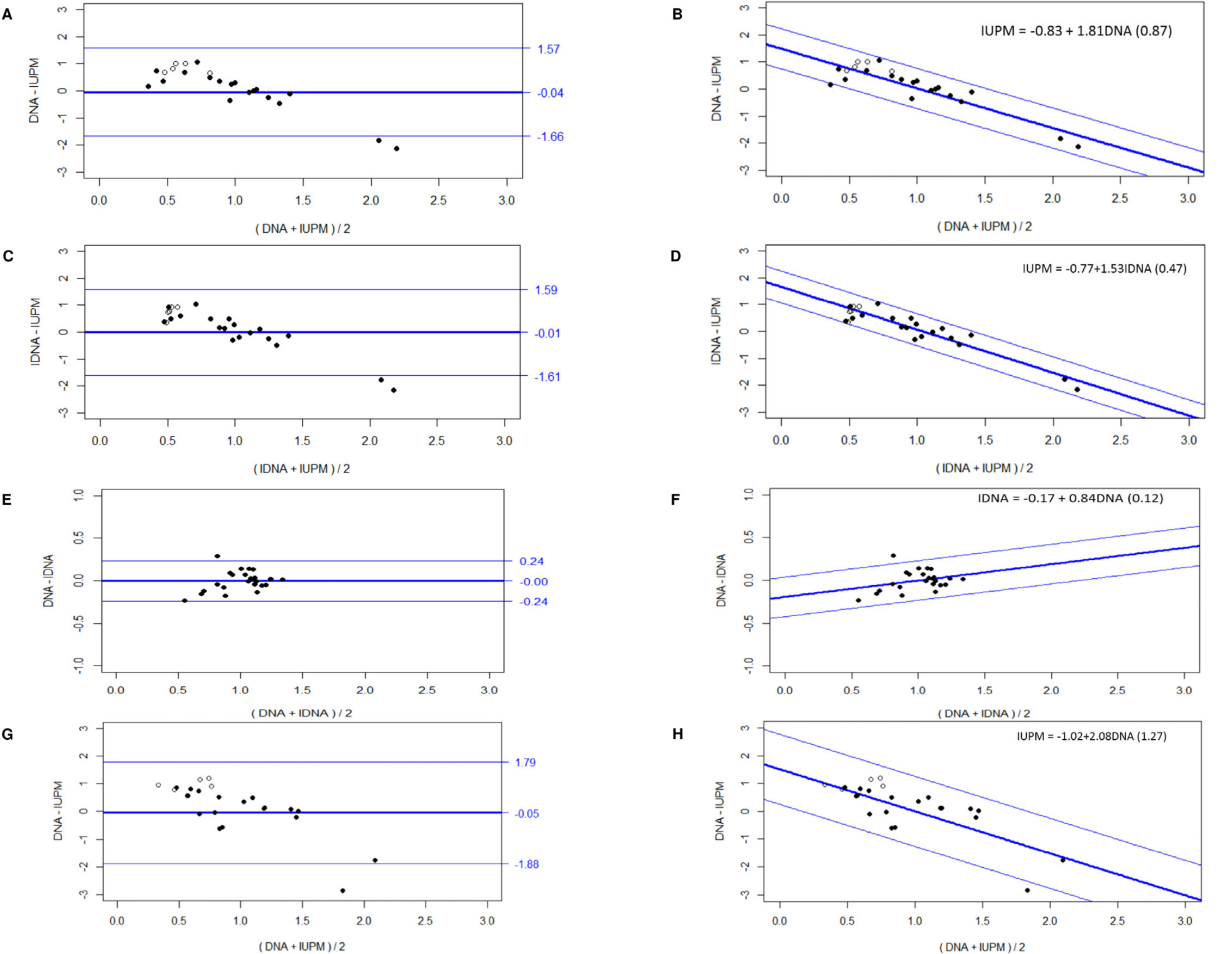
Bland Altman analysis. Agreement for HIV-1 copies quantification between: A) Total HIV-1 DNA (IDNA) and IUPM, mean difference (log _10_) and 95% Limits of agreement; B) Slope-intercept equation for total HIV-1 DNA and IUPM; C) Integrated HIV-1 DNA (DNA) and IUPM, mean difference (log _10_) and 95% Limits of agreement; D) Slope-intercept equation for integrated HIV-1 DNA and IUPM; E) Total HIV-1 DNA (DNA) and integrated HIV-1 DNA (IDNA), mean difference (log _10_) and 95% Limits of agreement; F) Slope-intercept equation for total and integrated HIV-1 DNA; G) Total HIV-1 DNA (DNA)–time point 1 and IUPM–time point 2, mean difference (log _10_) and 95% Limits of agreement; and H) Slope-intercept equation for total HIV-1 DNA–time point 1 and IUPM–time point 2. *Of note: Open label symbol represent test results below the detection threshold for VOA.

Bland Altman analysis revealed a mean difference (bias) between total HIV-1 DNA and integrated HIV-1 was median (95% limits of agreement) of -0.57 log_10_ (0.05 –(-1.19)). The mean quantification difference between the total HIV-1 DNA or HIV-1 copy numbers quantified with Alu-PCR (i.e. integrated HIV-1 DNA) and the VOA was 2.23 log_10_ and 2.88 log_10_, respectively. Because of this large difference, we normalized all data to the mean to enable a comparison by parametric statistics (Fig 4A–4F). The bias between total and integrated HIV-1 DNA was constant (Fig 4E). However, the bias between total or integrated HIV-1 DNA and the VOA was not constant (Fig 4A and 4C), but it clearly increased linearly with an increasing average viral reservoir size (Fig 4B and 4D). A regression analysis was performed using the quantification values of the methods (total and integrated HIV-1 DNA and VOA) to calculate the intercept and slope (Fig 4B, 4D & 4F). The corresponding intercept and slope for total HIV-1 DNA and VOA were -0.83 and 1.81 respectively. And the corresponding intercept and slope for integrated HIV-1 DNA and VOA were -0.77 and 1.53, respectively.

We also performed the Bland Altman analysis between the total HIV-1 DNA measurement from time point 1 and the VOA (median 2.5 years), to assess whether there is also a non-constant quantification bias. Indeed, we observed similar findings (Fig 4G and 4H) compared to the analysis between total HIV DNA and VOA from the same time point (time point 2) (Fig 4A and 4B).

### HIV-1 restriction factor gene expression

**Fig 5:**
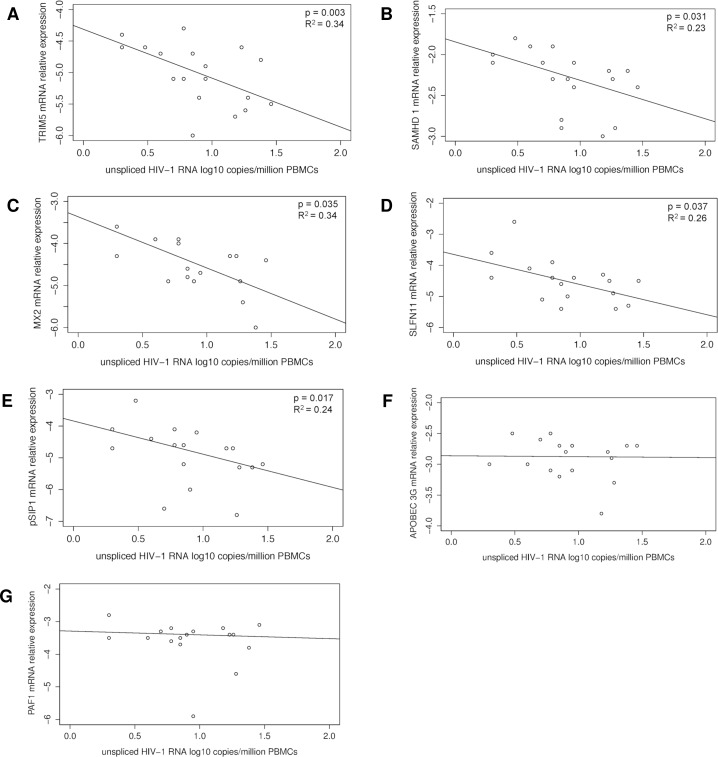
Linear regression analysis between unspliced (us) HIV-1 RNA and restriction factors. Associations between us HIV-1 RNA and: A) TRIM5α; B) SAMHD1; C) MX2; D) SLFN11; E) pSIP1; F) APOBEG3G; and G) PAF1.

Relative mRNA expression of 6 different restriction factors in CD4 T cells was measured at the second time point. Inverse significant association was found between cell-associated unspliced HIV-1 RNA and the following restriction factors: TRIM5α (R^2^ = 0.34, *p* = 0.003); SAMHD1 (R^2^ = 0.23, *p* = 0.031); MX2 (R^2^ = 0.34, *p* = 0.035); SLFN11 (R^2^ = 0.26, *p* = 0.037) and pSIP1 (R^2^ = 0.24, *p* = 0.017) (Fig 5A–5E). APOBEC3G and PAF1 were not correlated with levels of unspliced HIV-1 RNA, (*p* = 0.95, *p* = 0.79), respectively (Fig 5F–5G). In addition, a stepwise multivariate model was build, which confirmed the inverse significant associations between unspliced HIV-1 RNA and TRIM5α, SLFN11 and PSIP1 (p = 0.023, p = 0.035, p = 0.044, respectively).

## Discussion

HIV-1 cure strategies targeting the latent HIV-1 reservoir are increasingly being explored [19]. This emphasizes the need for relevant markers that can be used to monitor the replication competence of the viral reservoir and to assess efficacy of novel anti-latency strategies. Here, we performed a comprehensive evaluation of HIV-1 persistence markers in a representative patient cohort of long-term treated patients who started ART during chronic HIV-1 infection. We performed a longitudinal analysis of HIV-1 persistence markers measured by ddPCR and compared to the amount of replication-competent virus measured by VOA. In addition, this data was correlated with mRNA expression levels of host restriction factors.

In line with earlier reports, our study confirms the long-term stability of HIV-1 DNA levels in patients followed longitudinally [6, 20]. A stable copy number was observed for the cell-associate HIV-1 RNA, as well. A decrease on long-term was observed for only for 2LTR circles. There have been opposing results published for the half-life of 2LTRs *in vivo* [21, 22]. Also, it has been shown that changes in 2LTR circles were not associated with decreases in viral plasma RNA [23]. In our study, all patients were long-term treated with continuous viral suppression and did not show signs of productive infection during the study conduct. This may result in a decay of the 2-LTRs, which was observed.

It is well known that this latent reservoir is not eliminated despite continuous ART [24]. Homeostatic proliferation of infected cells and/or residual replication in lymphoid tissue compartments may contribute to HIV-1 persistence [25, 26]. The long-lived latent reservoir is exclusively composed of latent proviral HIV-1 DNA. However, most of the integrated HIV-1 DNA is not replication-competent. Therefore, if the total pool of integrated HIV-1 DNA fails to correlate with the relatively small replication-competent reservoir then HIV-1 DNA may not represent a good biomarker for monitoring viral persistence. In this study, a correlation was observed between the VOA and the levels of total and integrated HIV-1 DNA. This is the first study to report these correlations in one patient cohort. A correlation between VOA and integrated or total HIV-1 DNA was previously observed by Eriksson et al., [12] and Buzon et al., [16], respectively. This indicates that a stable fraction of integrated HIV-1 is replication-competent in patients treated during chronic HIV-1 infection. Moreover, we observe similar mean quantification difference between VOA and total and integrated HIV-1 DNA as was previously reported by Eriksson et al., (12).

Notably, in this study, Bland Altman analysis revealed a non-constant, but linear quantification bias between PCR based methods (for total and integrated HIV-1 DNA) and VOA in patients (Fig 4A–4F). Thus, indicating that both the total and the integrated HIV-1 DNA measures can predict the VOA quantification using a prediction formula based on the non-constant difference of the methods [27]. This mutual predictability in HIV-1 quantification between PCR based and cell culture based methods has not been reported yet and should be corroborated in further studies. Notably, our study only assessed patients on long-term ART, treated during chronic HIV infection. Future studies will be required to assess whether anti-latency drugs affect this correlation, as these treatments may only eliminate the replication-competent reservoir and may leave the total pool of HIV-1 DNA unchanged.

Interestingly, two recent treatment interruption trials revealed a correlation between integrated ant total HIV-1 DNA levels and time to viral rebound [9, 10]. Moreover, longitudinal decreases in HIV-1 DNA levels were observed in a substantial part of the functionally cured patients from the VISCONTI cohort [28]. Although a transient decrease in total HIV DNA, was recorded in a study where panobinostat was used to reactivate latent HIV-1 reservoir, no reduction in total HIV DNA, integrated HIV DNA, or infectious units per million cells were recorded for the whole cohort [29]. Despite, theoretical drawbacks, current clinical data indicate that HIV-1 DNA may still form a valuable biomarker for HIV-1 persistence. Therefore, PCR based assay quantifying integrated and/or total HIV-1 DNA should still be used in addition to viral outgrowth assays in future studies aiming at HIV-1 eradication [30, 31].

The measurement of integrated HIV-1 DNA by Alu-PCR [17] is of particular interest because stable HIV-1 reservoirs in blood have been found to mainly consist of resting CD4+ T cells harbouring integrated HIV-1 DNA. Although total HIV-1 DNA estimates may be biased by unintegrated HIV-1 DNA, the correlation observed in our study indicates that total HIV-1 DNA could be a good surrogate maker for integrated HIV-1 DNA in patients on stable ART as reported earlier [18].

### HIV-1 restriction and host factors

In this study a negative significant association was observed between unspliced HIV-1 RNA and several restriction factors (TRIM5α, MX2, SLFN11, SAMHD1, pSIP1). Only, three of these findings were confirmed in a multivariate analysis (TRIM5α, SLFN11 and PSIP1). This result could have been affected from the limited sample size used for this study. TRIM5α is known to directly limit HIV-1 production, by inhibiting viral transcription, and other members of TRIM family are found to have elevated expression levels in HIV elite controllers [32]. Similarly, in this study, higher expressed levels of TRIM5α were associated with lower unspliced HIV-1 RNA. SLFN11 exert its HIV-1 antiviral activity indirectly by depleting critical cellular resources for HIV-1 replication. Additionally, it is an HIV post-integration restriction factor, which may impede viral production, consistent with our observation between the expression of SLFN11 and unspliced HIV-1 RNA in patients on stable ART.

A negative association between the expression levels of PSIP1 and unspliced HIV-1 RNA was observed as well. It is known that PSIP1, which codes for LEDGF/p75 and p52 proteins, has been implicated in transcriptional regulation and mRNA splicing in vitro, however, its function in vivo is poorly understood. Recently, was shown that PSIP1 impairs the sites of HIV-1 integration in the host genome. In this study no association was found between integrated HIV-1 DNA and PSIP1 expression levels.

A significant inverse correlation was found in the univariate analysis between the expression of MX2 and SAMHD1 with unspliced HIV-1 RNA. MX2 is known to be a key effector of IFNα mediated HIV-1 resistance [33], and it has been suggested that it destabilizes nuclear forms of HIV-1 DNA [34]. Recently, it was proposed that MX2 may also interfere with post-nuclear trafficking and integration [35]. In this study we did observe a negative correlation between expression on MX2 and unspliced HIV-1 RNA, however, we did not find a significant correlation with the total or integrated HIV-1 DNA. As for SAMHD1, it is proposed that this factor may also restrict HIV-1 through degradation of viral single stranded RNA and/or DNA [36], we observed an association with unspliced HIV-1 RNA.

Intriguingly, no correlation was observed between APOBEC3G with any other HIV-1 reservoir markers quantified. Association between HIV-1 reservoir markers and differences in expression levels for APOBEC3G are reported for HIV-1 controllers [37, 38]. The current study was performed only with non HIV-1 controllers and no correlations with HIV-1 reservoir markers were observed. The PAF1 complex has not been fully characterized yet, although it has been postulated to block HIV replication during the early events from post entry to integration of proviral DNA into host genome. In this study, we did not observe an association between PAF1 and the HIV-1 reservoir markers [37].

The expression profile of host restriction factors may play an important role in determining markers of HIV-1 persistence and their dynamics in patients receiving suppressive therapy. Therefore, an evaluation of restriction factors with different mechanism of HIV-1 control may be informative in HIV-1 treatment studies.

### Conclusion

Our study reveals important correlations between the VOA and total and integrated HIV-1 DNA. These results show that the total pool of HIV DNA is correlated with the amount of replication-competent virus. Moreover, the quantification bias between the pool of HIV DNA and the viral outgrowth assay is functionally dependent, thus, indicating its predictive relevance at a single patient level. The correlation of HIV-1 RNA transcription and host restriction factors indicates that host restriction and co factors may be promising additional tools to monitor patients for functional cure studies.

## Materials and Methods

### Study population

Eligible patients were HIV-1 positive adult male and female participants from a previous study conducted at Ghent University Hospital [39]. All participants in the current study were receiving stable ART, reporting >95% adherence, and showing a plasma viral load <50 copies/ml for at least one year. Recruitment occurred at the time of routine visits at Ghent University Hospital. Patient selection was performed on the basis of baseline (i.e. time point 1) viral reservoir measurement (total HIV-1 DNA, cell-associated HIV-1 RNA and 2LTR circles), which were quantified in a previous study [39]. Three distinct groups of patients were recruited: 1) Patients with small HIV-1 burden; defined by total HIV-1 DNA—below 100 copies/10^6^ PBMCs, and the CA HIV-1 RNA—below 10 copies/10^6^ PBMCs (N = 10); 2) Patients with high HIV-1 burden; where the total HIV-1 DNA above 500 copies/10^6^ PBMCs, and the CA HIV-1 RNA is above 20 copies/10^6^ PBMCs (N = 10); and 3) Patients with discordant measures for total HIV-1 DNA and CA HIV-1 RNA; meaning low number of HIV-1 DNA copies and high number of CA HIV-1 RNA, and vice versa (N = 5).

Using the clinic’s electronic medical records database, additional information was collected: age, gender, current and nadir CD4+ T cell count, CD4/CD8 ratio, HIV-1 subtype, pre-ART plasma viral load zenith, total ART duration, and total duration of plasma viral load suppression <50 copies/ml. Whole blood was collected in 12 x 9 ml EDTA tubes at time point two, and additional blood draw of 6 x 9 ml EDTA tubes was taken after median (IQR) of 31 (28–36) days of the previous blood collection (i.e. time point 3). Fig 1 represents the complete workflow.

### Ethics statement

Ethical approval was obtained from the Ethics Committee of Ghent University Hospital (number: 2014/0545). All participants provided written informed consent.

### Sample preparation for intracellular HIV-1 DNA and HIV-1 RNA detection

Peripheral blood mononuclear cells (PBMCs) were isolated by Lymphoprep centrifugation using (ELITech Group, Benelux), re-suspended in fetal calf serum (FCS) and aliquoted in two parts of which one was stored at -80°C (to quantify PCR based reservoir markers) and the other in liquid nitrogen (for the VOA), until further processing. Total HIV-1 DNA, unspliced and multiply spliced HIV-1 RNA, and 2-LTR circles were quantified in PBMCs using the QX100 droplet digital PCR system (ddPCR) as previously described [40, 41]. Integrated HIV-1 DNA was quantified in PBMCs using Alu-PCR method as previously described [17].

To measure total HIV-1 DNA, nucleic acid was extracted from 5–10 x 10^6^ PBMCs using the DNeasy mini kit (Qiagen, Venlo, The Netherlands) with an elution buffer preheated at 56°C for 5 min. The isolated DNA was subjected to restriction digest by Promega EcoRI (Promega, Madison, Wisconsin) to ensure a uniform DNA distribution in the ddPCR droplets. Each restriction digest contained median of 2.8 μg (IQR 2.3–3.9) of DNA in a total volume of 16 μl, which was incubated at room temperature for 2h prior to the ddPCR assay.

To measure HIV-1 RNA, nucleic acid was extracted from 10–20 x 10^6^ PBMCs using the RNeasy mini kit (Qiagen), eluted in 30 μl nuclease-free water (Qiagen), and subjected to DNase treatment on column with the RNase free DNase kit (Qiagen). Total of 2 μg RNA was reverse transcribed using the iScript cDNA Synthesis Kit (Bio-Rad) and the cDNA was used to measure unspliced and multiply spliced HIV-1 RNA in the ddPCR assay.

To measure 2-LTR circles, nucleic acid was extracted from 5–10 x 10^6^ PBMCs using the QIAprep Spin Miniprep kit (Qiagen). Prior to extraction, samples were spiked with known copy numbers of the pSIF1-H1-Puro non-HIV plasmid (System Biosciences, Mountain view, California) as an internal control for normalization to cell equivalents per sample as described previously [40].

### Droplet digital PCR (ddPCR)

The ddPCR assays for measuring total HIV-1 DNA, unspliced and multiply spliced HIV-1 RNA, and 2-LTR circles were performed as reported previously using the described primer and probe sets, all primer pairs sequencing are provided in S1 Table. The ddPCR reaction mix consisted of 10 μl 2x ddPCR super mix for probes (Bio-Rad), 500 nM primers, 250 nM probe, and 4 μl of cDNA for HIV-1 RNA; 4 μl of restriction digest for HIV-1 DNA; or 4 μl of sample for 2-LTR circles, into a final volume of 20 μl. All samples were tested in triplicate; the reference gene—RPP30, was tested in duplicate. No-template controls (NTCs) with water and cDNA or DNA from non-infected PBMCs were included in every run. Results are reported as copies/10^6^ PBMCs, and for the changes in of the reservoir markers we have normalized to percentages of CD4 T cells. Inter assay coefficient of assay variation (CV) and test-retest CV (i.e. intra patient variation) were calculated for all ddPCR assays.

### Alu PCR (Integrated HIV-1 DNA)

Alu-HIV PCR was performed as described earlier by De Spiegelaere et al., [17] using 42 replicate reactions of a mix with gag-reverse HIV primer and Alu-forward primer as well as 42 replicates by use of only primers specific to HIV gag (HIV-only PCR). Samples were diluted to 2 or 10 μg DNA/mL and distributed in replicate PCR reactions containing 25 μL sample combined with 25 μL master mix, resulting in an equivalent of approximately 7500 cells (1 ng/μL PCR mix) or approximately 37 500 cells (5 ng/μL PCR mix) per 50 μL PCR replicate. All patient samples were run at 7500 cells/replicate PCR. We conducted the final nested qPCR in 20 μL with 10 μL of the first PCR product. This qPCR was optimized to enable robust amplification in a 1:1 dilution of PCR without PCR inhibition from pyrophosphates. We used master mixes and cycling conditions as previously described [42], and primer pairs are depicted in S1 Table. PCR cycling was performed on a Roche Real-*Time* PCR System (Light Cycler 480), and Cq values were obtained by second derivative analysis.

### Reference gene normalization for intracellular HIV-1 RNA quantification

To minimise assay variability, ddPCR results for the unspliced and multiply spliced HIV-1 RNA expression were normalized using internal reference genes [43, 44]. A set of eight reference genes was first ranked based on expression stability using the geNorm method in qBase Plus (Biogazelle, Ghent, Belgium) using real time quantitative PCR (qPCR) as previously described [45]. The geometric mean of the two most stably expressed reference genes (B2M and YWHAZ) was used to calculate the normalization factor of each clinical specimen [46]. Subsequently, raw ddPCR values for unspliced and multiply spliced HIV-1 RNA were normalized and reported as copies/10^6^ PBMCs for each patient, and for the changes in of the reservoir markers are normalized to percentages of CD4 T cells.

### mRNA expression of host/restriction factors

In all patient samples, mRNA levels of 7 restriction factors were assessed by quantitative reverse transcription PCR (RT-qPCR). TRIM5α, SAMHD1, APOBEC3G, MX2, PAF1, SLFN11, and pSIP1 were selected from previously published studies [32, 37]. The restriction factors were quantified by use of predesigned PrimePCR Sybrgreen Assays, from Bio-Rad (Bio-Rad, Hercules, California) with following unique assay IDs: 0010567, 0042858, 0048756, 0014994, 0003366, 0034707, and 00367525, respectively. qPCR concentration mix and cycling conditions were used as recommended by the manufacturer on a Roche Real-*Time* PCR System (Light Cycler 480). The raw qPCR data for the restriction factors were normalized with the 2 most stable reference genes as indicated for the HIV-1 quantification.

### Viral outgrowth assay (VOA)

Viral outgrowth assays were performed as previously described [16]. PBMC samples were shipped on dry ice to the Ragon institute (Cambridge, MA, US), total pool of CD4+ T cells were isolated from 100–150 million PBMCs and seeded at 20–50,000 cells/well in round-bottom 96-well plates. CD4+ T cells were stimulated with PHA (2μg/ml), rh IL-2 (100 units/ml) and co-cultured with irradiated allogeneic PBMCs from HIV negative healthy donors. At day 2 and day 9 of culture MOLT4/CCR5 cells were added to each well to enhance the survival of the cultured primary isolated cells and to support and facilitate further HIV-1 replication. Culture medium containing rh IL-2 (NIH AIDS reagents program) was replaced at days 2 and 6 of culture. After 12 days of culture, the cell supernatant from each well was harvested and incubated for 48h with TZM-bl cells (NIH AIDS reagents program), a permissive HeLa cell clone that contains HIV-1 Tat-responsive reporter genes for firefly luciferase under control of the HIV-1 LTR, permitting sensitive and accurate measurements of HIV-1 infection [16]. Luciferase activity was quantified by luminescence that is directly proportional to the number of infectious virus particles present in the initial inoculum. Estimated frequencies of cells with replication-competent HIV-1 were calculated using limiting dilution analysis, using http://bioinf.wehi.edu.au/software/elda/, as previously described [16].

### Statistical analysis

Baseline patient characteristics were described using either frequency for categorical variables or median [interquartile range (IQR)] for continuous variables. Differences between time points for the viral reservoir markers were assessed by standard non-parametric tests; Mann-Whitney and Kruskal-Wallis tests were used when appropriate. Linear regression and Spearman’s correlation were performed to assess relationships between different HIV reservoir variables. We report the adjusted R^2^ from the linear regression and the correlation coefficient as well. Linear regression analysis was performed to assess relationship between unspliced HIV-1 RNA and several HIV restriction and host factors, individually. In addition, a multivariate linear stepwise model was build. Bland Altman tests were used to assess the quantitative agreement between Alu-PCR (integrated HIV-1 DNA), the viral outgrowth assay (VOA) and total HIV-1 DNA (ddPCR) for HIV-1 copy measurements in patient samples. For all tests used, the level of significance accepted was *p*<0.05. Statistical analyses were performed using *R*, packages used MethComp and JAGS 3.4.0.

## Supporting Information

S1 Table Primer pairs used for markers quantification of persistence HIV-1 (XLSX)

S1 Fig Test-retest variability within patients for quantification of: A) unspliced HIV-1 RNA; B) multiply spliced HIV-1 RNA; C) Total HIV-1 DNA; and D) 2LTR circles. (TIFF)
