# Correction: Integrated and Total HIV-1 DNA Predict *Ex Vivo* Viral Outgrowth

**DOI:** 10.1371/journal.ppat.1005532

**Published:** 2016-03-22

**Authors:** Maja Kiselinova, Ward De Spiegelaere, Maria Jose Buzon, Eva Malatinkova, Mathias Lichterfeld, Linos Vandekerckhove

The funding section is incorrect. The correct funding statement is as follows: This work was supported by the Special Research Grant—BOF grant of Ghent University (01N02712); Research Foundation Flanders (FWO) (1.8.020.09.N.00); King Baudouin Foundation (2014-J1820640-201991); and the European Strategic Research Agenda for HIV/AIDS- HIVERA (B/14167). MK received travel grant for long stay abroad (V427314N) from the Research Foundation—Flanders (FWO). WDS is a post-doctoral fellow at FWO, grant number 12G9716N. LV is a Senior Clinical Investigator at the FWO, grant number 1802014N.MLis supported by NIH grants AI106468 and AI098487. The funders had no role in study design, data collection and analysis, decision to publish, or preparation of the manuscript.
