# Supplementary figures and images for: Integrated and Total HIV-1 DNA Predict Ex Vivo Viral Outgrowth
Source: PLoS Pathog. 2016 Mar 3;12(3):e1005472. doi: 10.1371/journal.ppat.1005472 (PMC4777389; doi:10.1371/journal.ppat.1005472)

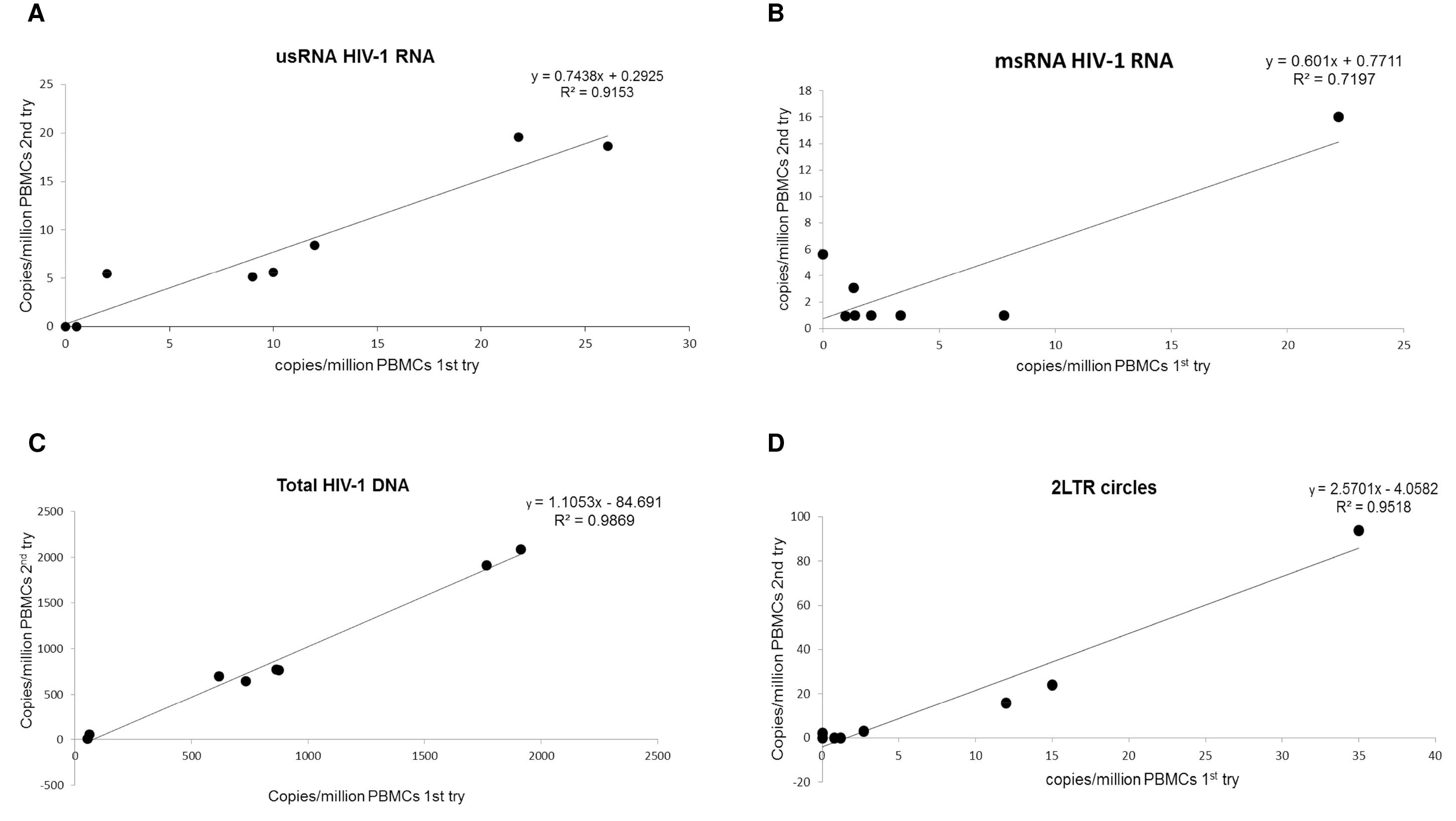

Supplement: S1 Fig — (TIFF) [file ppat.1005472.s002.tiff]
